# Pathological Targets for Treating Temporal Lobe Epilepsy: Discoveries From Microscale to Macroscale

**DOI:** 10.3389/fneur.2021.779558

**Published:** 2022-01-07

**Authors:** Jing You, Haiyan Huang, Clement T. Y. Chan, Lin Li

**Affiliations:** ^1^Department of Biomedical Engineering, University of North Texas, Denton, TX, United States; ^2^Department of Nutrition and Food Science, Texas Women University, Denton, TX, United States; ^3^Department of Neurology, University of California, Los Angeles, Los Angeles, CA, United States

**Keywords:** temporal lobe epilepsy, pathology, epileptogenesis, etiology, pathogenesis

## Abstract

Temporal lobe epilepsy (TLE) is one of the most common and severe types of epilepsy, characterized by intractable, recurrent, and pharmacoresistant seizures. Histopathology of TLE is mostly investigated through observing hippocampal sclerosis (HS) in adults, which provides a robust means to analyze the related histopathological lesions. However, most pathological processes underlying the formation of these lesions remain elusive, as they are difficult to detect and observe. In recent years, significant efforts have been put in elucidating the pathophysiological pathways contributing to TLE epileptogenesis. In this review, we aimed to address the new and unrecognized neuropathological discoveries within the last 5 years, focusing on gene expression (miRNA and DNA methylation), neuronal peptides (neuropeptide Y), cellular metabolism (mitochondria and ion transport), cellular structure (microtubule and extracellular matrix), and tissue-level abnormalities (enlarged amygdala). Herein, we describe a range of biochemical mechanisms and their implication for epileptogenesis. Furthermore, we discuss their potential role as a target for TLE prevention and treatment. This review article summarizes the latest neuropathological discoveries at the molecular, cellular, and tissue levels involving both animal and patient studies, aiming to explore epileptogenesis and highlight new potential targets in the diagnosis and treatment of TLE.

## Introduction

According to the updated version of the International League Against Epilepsy (ILAE) Classification of the Epilepsies, epilepsy can be classified into focal, generalized, combined generalized and focal, and unknown (1).

Temporal lobe epilepsy (TLE), the most common type of focal epilepsy, can be further classified into mesial TLE, involving the medial or internal structure, and neocortical or lateral TLE, involving the outer portion of the temporal lobe (neocortex) (2). Etiology of TLE varies between individuals. Structural, genetic, infectious, metabolic, and immune factors, singly or in combination, can contribute to the epileptogenesis in TLE. TLE includes the most medically intractable cases in epilepsy and is most frequently modeled to explore the mechanism of epileptogenesis. Some pathological features are evident in TLE, such as hippocampal sclerosis (HS) and cerebral lesions, whereas others are cryptogenic but identifiable, which remain the most challenging to diagnose (3). In this review article, we discussed the classical pathological features of TLE and summarized the newly promoted pathophysiological features in TLE, both from clinical and experimental research.

## Classical Pathological Features

Temporal lobe epilepsy (TLE) is clinically characterized by the progressive development of spontaneous recurrent seizures from the temporal lobe. Hippocampal sclerosis and focal cortical dysplasia (FCD) are important structural etiologies of TLE (4). Other brain lesions, including tumors and vascular malformations will not be discussed here.

### Hippocampal Sclerosis

Hippocampal sclerosis (HS) is the most common histopathological finding in TLE, comprising approximately 56–70% of intractable TLE cases in adolescents and adults (5, 6). Typical features of HS include neuronal loss and gliosis affecting CA1 and CA4 (or hilus) (7). The insult causing HS is still unclear, but it may be related to febrile seizures in early childhood (8).

The association between HS and chronic seizures is well-established, but there is a longstanding debate as to whether HS is a cause or consequence of chronic seizures (9).

**Figure 1:**
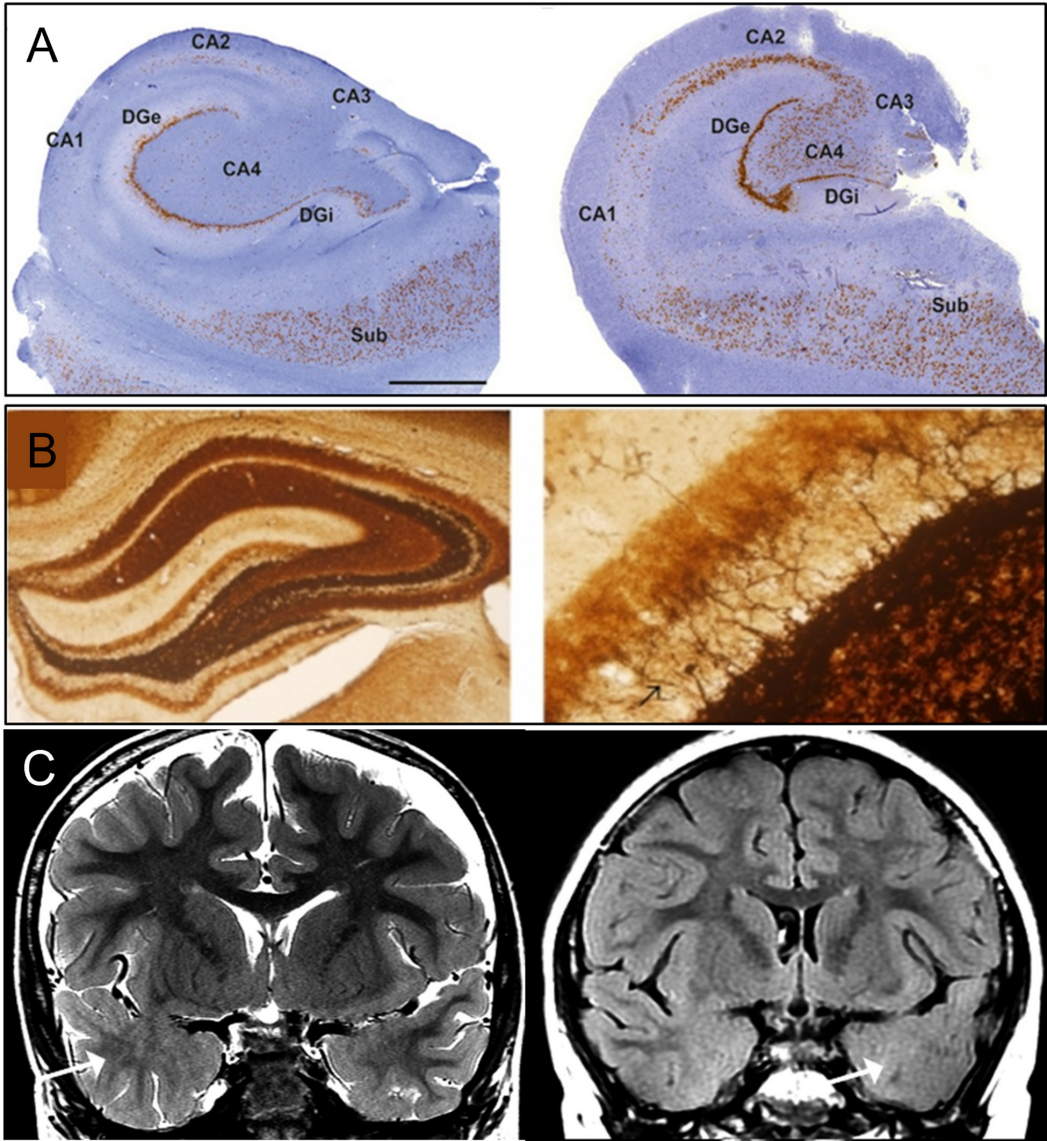
Classical pathology of temporal lobe epilepsy. **(A)** Histology images of hippocampal sclerosis (HS) in patients with temporal lobe epilepsy (TLE), type 1 (left) and type 2 (right) (10). In type 1, cell loss in both CA4 and CA1 can be observed. Damage to CA3 and CA2 is also visible. Granule cell loss happens in the internal limb of dentate gyrus (DGi) with cell preservation in the subiculum (SUB). In type 2, neuronal loss primarily involving CA1. **(B)** Mossy fiber sprouting under Timm's stain in KA-induced epileptic seizure rats (11), 40X (left) and 400X (right) with back arrow pointing to mossy fiber. **(C)** MRI imaging of focal cortical dysplasia (FCD) in two patients with TLE (12). Coronal T2-weighted imaging (left), and coronal T2-fluid-attenuated inversion recovery (FLAIR) sequence (right) images through the temporal lobes demonstrating asymmetric hyperintense temporal white matter signal and regional obscuration of corticomedullary interfaces in the temporal tip representing cortical dysplasia (Arrow). Figures were modified with permission.

Hippocampal sclerosis (HS) is classified according to the degree of neuronal loss and gliosis. The International League Against Epilepsy (ILAE) reached a consensus on its classification system to implement a reproducible, semi-quantitative scale of neuronal deficits in hippocampal subregions (10). This classification depends on the patterns of neuronal loss and gliosis, regardless of fiber sprouting and neuronal alterations. HS type 1 is the most common type, accounting for 60–80% of TLE-HS cases. CA1 is the most severely affected area with cellular loss of >80%, whereas other less affected areas include CA2 (30–50% loss of pyramidal cells), CA3 (30–90% neuronal loss), and CA4 (40–90% neuronal loss). Almost 50–60% of granule cells are lost from the dentate gyrus (DG) in HS type 1 (Figure 1A). HS type 2 involves predominantly CA1 neuronal cell loss and gliosis, affecting almost 80% of pyramidal cells. In addition, there is a mild cell loss in CA2 (<20%), CA3 (<20%), and CA4 (<25%). No severe granule cell loss is observed in DG in type 2. HS type 3 involves predominant cell loss in CA4 (almost 50%) and DG (35% cell loss), whereas CA3 (<30%), CA2 (<25%), and CA1 (<20%) are moderately affected. Type 3 is more likely to result from dual pathology (10).

Besides prominent cell loss and gliosis, mossy fiber sprouting is another pathological characteristic of HS, which involves aberrant sprouting of granule cell axons (Figure 1B) (13). There are two phases of mossy fiber sprouting formation: phase 1 involves injury-induced neuronal activity and growth factors release (14–16); whereas Phase 2 involves the growth and extension of the granule cell axon (17–19). DG is morphologically formed by three layers: molecular, granule cell, and polymorphic layer (hilus). The molecular layer consists of apical dendrites of granule cells and excitatory terminals that communicate between the entorhinal cortex and the outer molecular layer or between the commissural projections to the inner molecular layer. The granule cell layer is formed by densely packed cell bodies with axons (mossy fiber) that extend to the hilus and project to excitatory interneurons (mossy cell). Under normal conditions, mossy fibers are infrequently seen in the molecular layer of hippocampal tissue, but the dense existence of zinc channels can be visualized with neo-Timm staining in TLE specimens (13).

Time-related factors can affect the extent of HS, such as age, seizure frequency, and seizure duration. Animal models have been widely used to reproduce the pathological features of HS observed in patients with TLE (20). In the intrahippocampal kainic acid model of mesial temporal lobe epilepsy (mTLE) (21), hippocampal neuronal loss and fiber sprouting play a critical role in the temporal lobe epileptogenesis. Diagnosis of TLE-HS in the early stage is of significance in seizure outcomes and psychological performance after resective surgeries. Through cross-validation in patient studies, the removal of neuronal deficient area within the hippocampus resulted in seizure free or reduced seizure frequency in many patients (22). During a mean follow-up of 38 months, 48 of 56 (86%) patients with TLE-HS are seizure-free after temporal lobectomy (23).

### Focal Cortical Dysplasia

Focal cortical dysplasia, first described in 1971, arises in the early stage of cortical development and is caused by abnormalities during neuroglial proliferation and differentiation, neuronal migration, and cortical reorganization (Figure 1C) (13, 24). Architectural abnormalities mainly include cortical laminar disorganization and columnar disorganization, which can be identified through histological testing (24, 25).

More severe FCD is characterized by the presence of balloon cells and abnormal neuronal elements, including immature neurons, dysmorphic neurons, and giant cells (25). Distinctive from FCD type I and type III, FCD type II is characterized by the presence of dysmorphic neurons or balloon cells (25, 26).

The clinical finding of FCD in patients with epilepsy implies a causal relationship of FCD to the development of epilepsy (27). Both an increased excitatory and a decreased inhibitory state are observed. Studies at the molecular level have suggested several potential mechanisms for FCD-induced hyperactivity. One mechanism involves altered expression of NMDA receptors–the excitatory glutamate receptors involved in calcium transportation (28). Numerous studies have revealed that FCD lesions are accompanied by an up-regulated expression of NR2A/B sub-unit proteins and their associated clustering protein PSD95 in the dysplastic neurons, which remain in a low expression level under normal conditions (29). Another glutamate receptor AMPA family is also involved in the pathogenesis of FCD. Elevated expression of AMPA subunits has been found in FCD lesions (30). However, the underlying mechanism leading to epileptogenesis remains unclear.

In addition to hyperexcitability, decreased inhibition is also observed in the pathogenesis of FCD. The absence of inhibitory GABAergic neurons in dysplastic lesions was further confirmed by experimental FCD model and dissected tissues from patients (31, 32).

## Gene-Related Pathology

The etiology of TLE can be genetic, acquired, or a combination of both. Genes involved in TLE development are still unclear. Herein, we suggest several possible candidates for reference.

### Schizophrenia-Related Gene DTNBP1

The prevalence of epileptic seizures in psychosis has recently attracted much attention. According to a retrospective cohort study, patients diagnosed with schizophrenia have a higher risk of epileptic seizures (33). Additionally, epileptic patients have an elevated risk of schizophrenia. This evidence suggests a shared mechanism between schizophrenia and epilepsy.

Owing to exon sequencing, a shared genetic pathway exists between the development of schizophrenia and epilepsy. Therefore, genes that promote schizophrenia may also play important roles in epilepsy. One such example is the DTNBP1 gene, which encodes the dystrobrevin-binding protein 1 that is involved in organelle biogenesis and plays key roles in brain development and neuronal excitability (34). Decreased expression of DTNBP1 can lead to reduced exocytosis of brain-derived neurotrophic factor from cortical excitatory neurons, which further limit the number of inhibitory synapses around excitatory neurons (35). Studies show that the hippocampus has high expression of DTNBP1 under normal conditions, but the levels of this protein in schizophrenia patients are significantly reduced, as supported by postmortem analysis (36). As the hippocampus is crucial in TLE pathology, experiments have been conducted to explore the genetic effects of DTNBP1 in TLE. Six candidate variations in DTNBP1 gene region were used, which were separately involved in hippocampal gray matter volume (rs2619522), gray matter and white matter volume (rs2619538), hippocampal glutamate concentration (rs760665 and rs909706), and volume reduction of multiple brain regions (rs1011313) (33). The variations of gene expression have been explored in TLE patients and pentetrazol (PTZ)-induced epileptic rat model. Among these variations, rs2619538 is located in the promoter region of DTNBP1 gene and its mutation is related to the up-regulation of DTNBP1 transcription. As rs2619538 is associated with reduced volume of gray matter and white matter in young children, DTNBP1 may play a key role in brain development and dysfunction; therefore, cortical dysplasia may cause early-onset seizures. Other evidence indicates that the DTNBP1 gene plays a role in the transportation of glutamate, which modulates excitatory and inhibitory changes of neuronal activity. These findings suggest that DTNBP1 is critical in the pathological development of epilepsy.

### MicroRNA Expression in Neocortex

**Figure 2:**
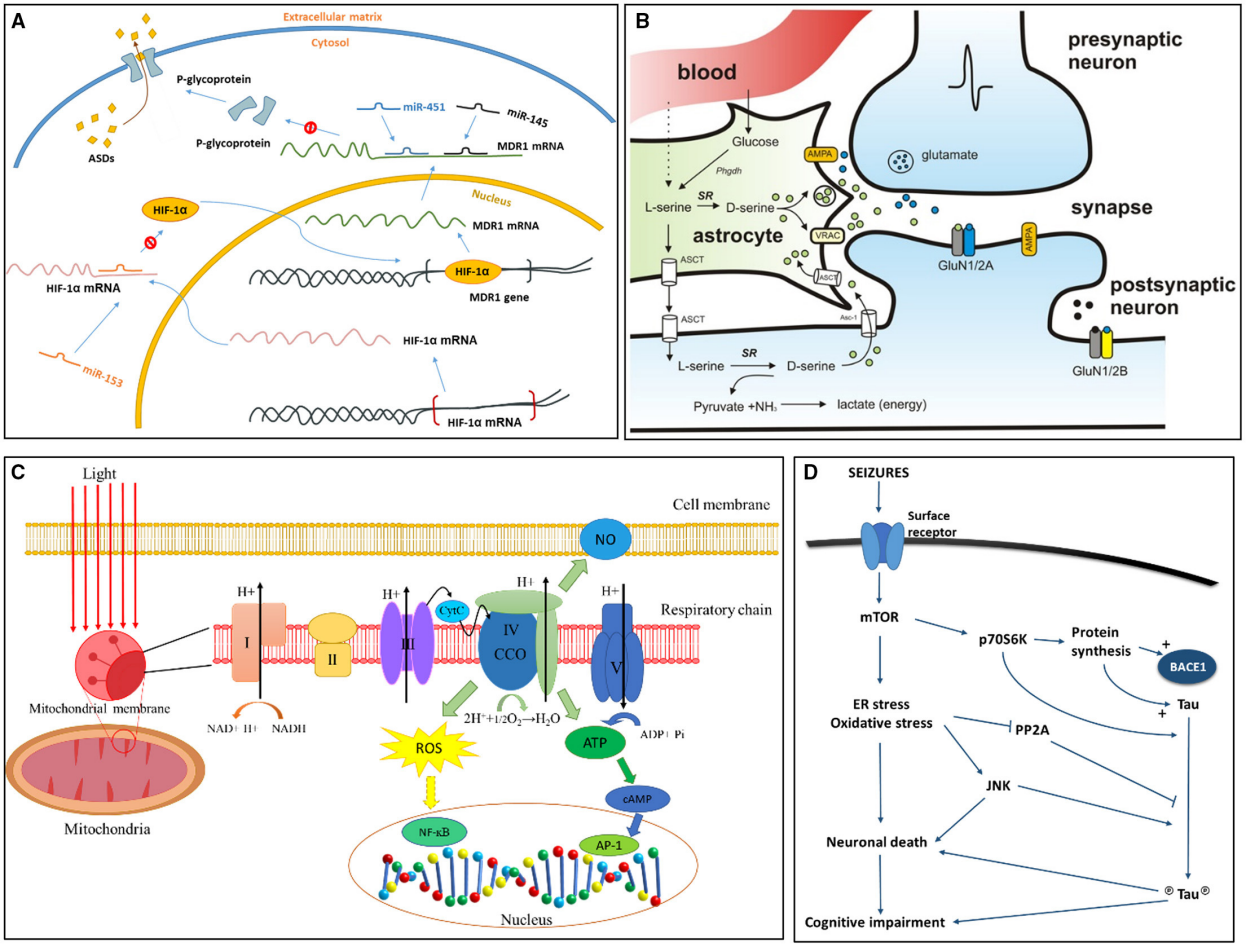
Signaling pathways more recently proposed to TLE epileptogenesis and associated conditions. **(A)** Role of miRNA in pharmacoresistant epilepsy (39). Binding of miR-153 blocks the translation of hypoxia-inducible factor 1α (HIF-1α) mRNA. Without limitation of miR-153, HIF-1α mRNA is translated, and HIF-1α protein transports to the nucleus, binding to the multidrug resistance gene 1 (MDR1) gene as a transcriptional regulator. When MDR1 mRNA enters cytoplasm, it translates into P-glycoprotein, which combines to the cell membrane pumping antiseizure drugs (ASDs) out of the cell. Existence of miR-145 and miR-451 can inhibit translation of MDR1 mRNA by binding to its 3' untranslated region, thus decreasing expression of P-glycoprotein. **(B)** D-serine synthesis and release pathway (40). Activation of presynaptic neurons induce the release of glutamate, which bind to AMPA receptors on neighboring astrocytes to release D-serine. D-serine is transformed from L-serine by serine racemase (SR). N-methyl-D-aspartate receptor (NMDAR)-containing GluN2A subunits are major binding sites of D-serine. Glycine competes with D-serine for the binding of GluN2B. **(C)** Reactive Oxygen Species (ROS) signaling in mitochondria (41). Respiratory chain exists on the inner membrane of mitochondria and is composed of five subunits (I, II, III, IV, V). Unit IV of respiratory chain is also known as cytochrome c oxidase (CCO), it reduces oxygen into water through electron transport. Electron transport further produces ATP and ROS. The oxidative stress associated with ROS will change the level of NF-kB, AP1, and cAMP, and these factors are actively involved in gene translation and expression and may result in cell death. AP1, activator protein 1; ATP, adenosine triphosphate; CCO, cytochrome c oxidase; CytC, cytochrome C; H^+^, hydrogen ions; cAMP, cyclic adenosine monophosphate; NF-kB, nuclear factor kappa B; NO, nitric oxide. **(D)** Hypotheses for tau pathology on cognitive impairment and epileptogenesis in TLE (42). Seizures induce the activation of surface receptors, involving excitatory neurotransmitter receptors, which further activates the mTOR pathway leading to an increase in endoplasmic reticulum (ER) stress and oxidative stress. These chronic stresses will result in neuronal death and subsequent cognitive impairment. p70S6K, a ribosomal protein kinase downstream of activated mTOR, stimulates the synthesis of tau and BACE1 proteins directly phosphorylates tau (circled P). Cellular stress also induce activation of pro-apoptotic JNK, and inhibiting PP2A activity. PP2A is a major tau phosphatase and can decrease tau phosphorylation upon activation. Figures were modified with permission.

MicroRNA (miRNA) is a single-strand endogenous non-coding RNA that downregulates posttranscriptional gene expression through binding to the 3′-UTR region of mRNA (37). These miRNA species are involved in the regulation of several physiological processes, such as proliferation, differentiation, metabolism, and apoptosis; the regulatory effect of miRNA plays an important role in nervous system disease development (38). It is clear that some miRNAs are related to epilepsy in animal models (Figure 2A). For example, miRNA-132 is the first of its kind to be demonstrated to cause seizure activity, based on a pilocarpine-induced mouse model (37). Additionally, miRNA-34a was linked to the apoptosis of hippocampal neurons in a rat TLE model, which may contribute to the development or maintenance of epilepsy (43). In a recent human study, a series of miRNA (miRNA-1260, 1275, 1298, and 451) were identified in the hippocampus of intractable mTLE with HS (44) and were linked to the neurotrophic signaling, axon development, K^+^ channel, and GABA receptor regulation (45). Another TLE patient study with ILAE HS type 1 reported seven dysregulated miRNAs, which are related to the pathways of nucleic acid binding, intracellular and cellular macromolecule metabolic processes, and the PI3K-Akt signaling pathway (46). Further research involving the analysis of miRNA levels in the neocortex of TLE patients showed that the expression profile of miRNA may vary depending on specific brain locations, some miRNAs were positively correlated with seizure frequency, and some were positively correlated to the use of antiepileptic drugs (37, 38, 47).

## Molecular-Related Pathology

In contrast to the gene-related pathology, which affects epileptogenesis through direct effects on gene transcription and expression, molecules cause epileptogenesis through binding to receptors to modulate downstream or upstream signaling pathways.

### D-Serine

N-methyl-D-aspartate receptor (NMDAR) mediates neuronal hyperexcitation, which represents a key step in epilepsy development. Therefore, regulation of NMDAR is a potential target for the treatment of epilepsy (48–50).

D-serine is a promising molecule for the regulation of NMDAR activities (Figure 2B) (51). It is an endogenous amino acid that is produced from L-serine by serine racemase (SR) (52). Previous studies of SR knockout mice and pilocarpine-treated rats confirmed its high abundance in neurons and astrocytes (53, 54). However, the exact role of D-serine is controversial. Some researchers proposed that D-serine prevents the loss of neurons in the medial entorhinal area (MEA) through inhibition of Ca^2+^-induced hyperexcitability by blocking highly calcium-permeable receptors and depletion of D-serine, as observed in MEA from epileptic animals (55). Other researchers argued that the interaction between D-serine and NMDARs can enhance the synaptic function and maintain long-term potentiation. This co-agonist role of D-serine has been confirmed by injecting an SR inhibitor to the lateral ventricles in a pilocarpine-induced rat model (56). With reduced D-serine production, these rats had alleviated seizure development with prolonged latencies and shortened duration of seizures. Similar results were also observed in SR knockout mice (57). Further evidence supports the essential role of D-Serine in the forebrain of epileptic animals. A reduced level of D-serine is observed in the forebrain, which is linked to cognitive dysfunction (58).

### High Mobility Group Box 1 (HMGB1)

The initial insult in TLE usually occurs with a trauma or an infection, which induces inflammation in the brain. A plethora of findings have highlighted the crucial pathophysiological role of brain inflammation in epileptogenesis (59–61). Emerging research on animals suggest that high mobility group box protein 1 (HMGB1), an initiator and amplifier of neuroinflammation, is a potential target for epilepsy treatment (62). HMGB1 is a nuclear protein ubiquitously released by glia and neurons in response to inflammation (63). It interacts with receptor for advanced glycation end products (RAGE) and toll-like receptor 4 (TLR4) on targeted cells. HMGB1 plays an important role in epileptogenesis through neuroinflammation and disruption of the blood brain barrier (BBB) (64–66).

High mobility group box protein 1 (HMGB1) responds to epileptogenic insults as an inflammatory cytokine. Therefore, it is immediately released into the extracellular space upon neuronal injury (67). Antagonists of HMGB1 and TLR4 inhibit seizure precipitation, reduce seizure frequency and duration, and prevent acute and chronic seizure recurrence both in mice and human epileptogenic tissue (68). By regulating the HMGB1/RAGE/TLR4 axis, the translocation of HMGB1 is inhibited, but the BBB integrity is maintained, as supported by antibody studies on pilocarpine-induced TLE animal model and TLE patient samples (69–71).

### Innate Immune Receptor TLR4

There is increasing evidence to support the indispensable role of neuroinflammation in epileptogenesis. Toll-like receptor (TLR) signal transduction is highly involved in the convulsive disorder, which serves as a good starting point for understanding the interplay between neuroinflammation and development of epilepsy. As a member of TLR family, TLR-4 has been reported to be actively involved in epileptogenesis through modulating neuronal excitability (72). TLR4 drives autoimmune response and regulates cytokine secretion and microglial phagocytic activity. Endogenous ligands of TLR4 include HMGB1 protein, heat shock proteins, and the myeloid-related protein eight. In some rodent studies, binding of TLR4 to the inflammatory stimulus, such as LPS-enhanced, KA-induced (73), and PTZ-induced seizure susceptibility (74), an increase in the levels of TLR4 ligands activated TLR4 and increased seizure duration and frequency (68, 75–81).

These studies imply that activation of the TLR4 signaling pathway is related to the pathogenesis of epilepsy. Thus, manipulating TLR4 and its endogenous ligands may suppress seizures, which may be a potential therapeutic strategy for epilepsy (68).

### Neuropeptide Y

Neuropeptide Y (NPY) is widely distributed in the central nervous system and is emerging as a new target for vector-applied gene therapy (82). Local NPY-immunoreactive neurons, fibers, and specific binding sites can be found in the DG and subiculum. Increased hippocampal expression of NPY has been reported in many models of induced seizures, such as electrical kindling and kainic acid-induced models (83, 84). Many studies have shown seizure-suppressing effects of NPY in the hippocampus of both rodents (84–88) and hippocampal slices from pharmacoresistant epilepsy patients (89–91). NPY elicits its biological actions in the brain mainly by binding to Y1, Y2, and Y5 receptors, members of a G-protein coupled receptor superfamily (92). In the hippocampus, the seizure-suppressing effects of NPY appear to be mediated primarily via activation of Y2 receptors (93), while Y5 receptors may also play a role particularly outside the hippocampus (86, 87, 94). In contrast, Y1 receptors appear to generate an opposite response (95–97). This effect of NPY on seizures is thought to occur through the activation of presynaptic NPY Y2 receptors (93, 98) that inhibit voltage-gated calcium channels (99), thereby reducing glutamate release probability in excitatory synapses.

## Cell-Related Pathology

Neurons are the minimum working units in the central nervous system. They carry signals from upstream regions and convey them downstream through the synapses. Structural changes, such as those involving the cytoskeleton and extracellular matrix, can affect synaptic stability and interfere with transmission of information between cerebral areas. Furthermore, synaptic transmission requires energy, which is adenosine triphosphate (ATP) supplied by mitochondria. Thus, mitochondria and related cell metabolism can be a potential target for epilepsy. The related pathological discoveries are discussed below.

### Cellular Metabolism: Mitochondria and Neuroprotection

A plethora of studies have suggested that mitochondrial dysfunction forms an integral part of the development of epilepsy (100–103). Mitochondria are the major generators of reactive oxygen species (ROS) in cells (104). The release of ROS damages elements in these organelles, such as mitochondrial DNA (mtDNA), mitochondrial membranes, respiratory chain proteins, and nuclear DNA, leading to mitochondrial dysfunction (Figure 2C) (105, 106). ROS overproduction-induced oxidative stress in mitochondria is a vital factor in epileptogenesis and seizure generation (107).

Augmented activities of enzymes such as glutathione peroxidase (GPx) and glutathione reductase (GR) in mitochondria provide neuroprotective effects against ROS-triggered oxidative damage in epileptic patients (108). Clinical data indicate that oxidative mitochondrial damage can be diminished by enhancing the activity of mitochondrial antioxidant enzymes. Antioxidative agents can be used to counteract mitochondrial oxidative stress. These agents include, but are not limited to, endogenous antioxidants, polyphenols, vitamins, thiols, and nuclear factor E2-related factor 2 (Nrf2) activators (109–114). Supporting evidence shows that antagonizing oxidative stress in mitochondria via antioxidants can attenuate or slow disease progression in a number of experimental epilepsy models (115, 116).

Furthermore, ROS can damage electron transport chain through oxidizing mtDNA, which is proposed to affect disease onset and the progression of mitochondrial disorders in humans (117). Excessive ROS-triggered oxidative stress due to mitochondrial dysfunction triggers altered mitophagy and promotes the development of neurological diseases. The brain is particularly susceptible to oxidative stress due to the high level of oxygen consumption and lipid-rich content (118). Thus, aberrant mitophagy (either uncontrollable or inadequate mitophagy) due to mitochondrial defects has a strong potential to initiate epilepsy.

Mitochondrial abnormalities have been observed in epileptic foci and nearby areas, while no mitochondrial pathology in the brain tissue has been investigated yet (e.g., in the parahippocampal gyrus of patients with clearly pronounced hippocampal pathology and a hippocampal seizure focus) (119). Clinical evidence further supports this claim: mitochondrial dysfunction in the hippocampal CA3 and pronounced drops in NAD(P)H fluorescence transients were observed in TLE patients (120). These findings were interpreted as supporting the hypothesis that the hypometabolism in the epileptic focus is more of a reflection of dysfunction in cellular energy metabolism rather than of neuronal cell loss. Enhancing antioxidant capacity of the mitochondrial compartment may hold promise for disease prevention and treatment (121).

### Microtubules

Synaptic connections are critical for conveying information in neural circuits and synaptic reorganization. The rearrangement of excitatory and inhibitory circuits has been reported to be associated with the occurrence of TLE. The postsynaptic density (PSD) is a huge protein complex, including receptors, scaffold proteins, signaling enzymes, and cytoskeletal proteins. PSD is located on the postsynaptic membranes of excitatory synapses and are crucial for synaptic transmission and plasticity (122).

As a major component in the cytoskeleton, microtubules (MTBs) are implicated in the modification and maintenance of cell morphology, along with the division and migration of cells, by interacting with microtubule-associated proteins (MAPs) (123). MTBs consist of α-tubulin and β-tubulin heterodimers and are in a dynamic state of assembly and disassembly, which is significant for its functions and implementation. Based on the dynamic instability of the MTB system, depolymerization and polymerization agents were used to explore the role of MTB in epileptogenesis. Phosphorylation and oxidation of MTBs were hypothesized to cause depolymerization, which further disintegrate the neurons. This evidence supports the critical role of MTBs in the synaptic function via change of cell morphology and protein composition.

With the confirmation of its abundant expression in the synaptic structure, MTBs are given extra attention for their promising role in the development of epilepsy (124, 125). In a pilocarpine-induced TLE rat model, colchicine was used as depolymerizer and paclitaxel as a polymerizer to explore the function of MTB (126). Both α-tubulin and β-tubulin were observed to be downregulated compared to the control group in the latent and chronic period (126). Together with the downregulation of MTBs, increased hippocampal neuronal loss was reported in epileptic rats. Additionally, paclitaxel reduced the chronic seizure occurrence rate and increased β-tubulin expression in the hippocampus. Furthermore, it has been observed that the recovery of MTBs occurred simultaneously with mossy fiber sprouting, indicating that these processes may involve the synaptic remodeling in epileptogenesis. MTBs may also contribute to the development of epilepsy through interacting with NMDAR and affecting Ca^2+^ influx (127, 128). MTBs may dysregulate the NMDAR expression and affect the plasticity of epileptic synapses after status epilepticus. Excessive Ca^2+^ concentration may accelerate the depolymerization of MTBs, and depolymerized MTBs may facilitate Ca^2+^ influx. This evidence suggests a promising role of MTBs in the prevention and treatment of refractory seizures.

### Extracellular Matrix

While cellular changes during the latent period have been widely explored, limited attention has been given to the extracellular space. Extracellular matrix (ECM) forms the extracellular structural base, and the reorganization of ECM has been linked to the development of epilepsy (129). The spatial revolution of ECM guides neurogenesis, migration, and axon growth during brain development. It also mediates synaptic plasticity and prevents aberrant synaptic remodeling in mature brains (130).

The extracellular matrix (ECM) can be remodeled by neurons using an array of gelatinases called matrix metalloproteinases (MMPs). MMPs are involved in the regulation of glutamatergic neurotransmission and hippocampal long-term potentiation (131, 132).

Among the MMP family, MMP9 and MMP2 are abundantly found in the brain and have been implicated in epileptogenesis based on assays from human epileptic tissues and epileptic animals (133). Thus, monitoring ECM remodeling can be helpful in elucidating epilepsy development (134). Using a gelatinase biosensor activatable cell-penetrating peptide (ACPP), researchers observed that ECM remodeling first appeared on pyramidal cells, suggesting that neurons are the primary source of MMPs through epileptogenesis.

In addition, the inhibitor of MMPs has been reported to have antiseizure and antiepileptogenic effects (135, 136). A BBB-permeable MMP inhibitor IPR-179 was used on two TLE rodent models: a rapid-kindling rat model and KA mouse model (132, 137, 138). This inhibitor has a high affinity for MMP9 and low affinity for MMP2 (139). In the rapid-kindling rat model, IPR-179, had antiseizure effects, because IPR-179–treated animals showed less severe behavioral seizures compared to vehicle-treated animals. This effect was maintained after inhibitor removal.

### Astrocytes

#### K^+^ Signaling

Astrocytes are the most common cell type in the brain. They not only provide energy to neurons but also participate in maintaining the ionic homeostasis in cells. When the neurons of the central nervous system are excited, the K^+^ level in the periphery of the cell significantly increases (140). The role of astrocytes is to reduce the K^+^ concentration, inhibit nerve impulses, and restore cells to ensure that they can be immediately excited again (141). Epilepsy is related to uncontrolled behavioral changes caused by excessive excitability of neurons. The function of astrocytes is to regulate the K^+^ concentration, which affects the onset of epilepsy.

Normal neuronal activity requires a rapid balance of K^+^ concentration inside and outside the cell, which involves two mechanisms: K^+^ uptake and spatial K^+^ buffering (142). The Kir channel in astrocytes is believed to have the hypothetical properties of K^+^ homeostasis and is used to study the connection with epilepsy (141). Studies have shown that when Kir channel expression is impaired, the K^+^ buffer in the hardened human hippocampus is impaired. Patch clamp analysis confirmed that decreased Kir channel expression can lead to K^+^ buffer disturbance and increased susceptibility to epilepsy in patients with mTLE-HS (143, 144). Kir4.1 is the subunit K^+^ channel of the astrocytes and has been shown to have significant losses in patients with mTLE-HS (145–147). Studies have reported that traumatic brain injury impairs K^+^ homeostasis, and the extracellular K^+^ concentration increases, leading to spontaneous partial seizures (148). An increasing number of studies have proved the importance of K^+^ homeostasis in the control of epilepsy in astrocytes, which will provide new insights for the treatment of epilepsy in the future.

#### Astrogliosis

Being the most common cell type in the brain, astrocytes are of great significance in energy metabolism, stabilization of neurotransmitters, and network formation in the brain (149, 150). Astrogliosis refers to a pathological increase in the number of astrocytes, producing behavioral and morphological changes. Astrogliosis is considered to be a pathological feature of medial brain TLE (151, 152). To further evaluate the pathogenesis of TLE and astrocyte proliferation, Lu et al. examined the changes in three proteins in astrocytes, including glial fibrillary acidic protein (GFAP), metallothionein (MT), and aquaporin-4 (AQP4), in patients with mTLE.

Glial fibrillary acidic protein (GFAP) is a cell-specific marker that can distinguish astrocytes from other cells. MTs are a family of cysteine-enriched proteins with low molecular weight. They can bind to zinc and cadmium in cells. MTs have four isoforms, which are endogenous expressed in various tissues. Isoforms I, II, and III are expressed in the mammalian brain, of which MT-I/II are mainly expressed in astrocytes (153, 154). Aquaporin is located on the cell membrane and can control the flow of water in and out of the cell (155). AQP4 is an aquaporin that is expressed in astrocytes (156). These three proteins are indicators of astrocytosis, and Lu et al. hypothesized that the expression levels of these proteins differ between mTLE patients and healthy people (157).

They recruited three groups of 30 patients with mTLE in the experimental group and five healthy people in the control group. Hippocampal samples from patients were cut into 50 μm sections for analysis. The researchers first used reverse transcription-polymerase chain reaction (RT-PCR), which showed that the expression levels of GFAP, MT-I/II, and AQP4 in patients with mTLE were significantly higher than those in the control group. They also found through immunofluorescence detection that the signals of these three proteins were higher in patients with mTLE than in the control group. GFAP, MT-I/II, and AQP4 are important indicators of astrogliosis. This study confirmed that the levels of these three proteins in patients with mTLE are higher than those in healthy people (157). These three protein indicators of astrocytosis can be used as entry points in the treatment of mTLE.

#### Glutamine

Glutamine synthetase (GS) is an enzyme that catalyzes glutamine by combining ammonia and glutamate (158). The cytoplasm of astrocytes contains a large quantity of GS, which participates in the metabolism of amines (159). When astrocytes lack GS, the metabolism of amines is reduced, which leads to an increase in the concentration of brain amines and subsequently induces seizures (160, 161).

Researchers have observed that patients with medically refractory epilepsy lack GS in astrocytes, and the immunoreactivity of GS is generally reduced (162). Haberle et al. reported three deaths due to mutations in the glutamine synthetase gene, two of which showed clinical signs of seizures during their lifetime (163–166). He et al. performed an experiment with GLUL knockout mice, which proved that a lack of GS can lead to increased susceptibility to seizures (167). The researchers injected methionine sulfoximine into the rat brain to inhibit GS in the medial temporal lobe and prevent the catalysis of glutamine (168). The experimental results confirmed that the focal inhibition of GS can induce epilepsy. Although the mechanism of epilepsy induced by GS is not clear, an increasing number of studies have confirmed the connection between them. Therefore, controlling the expression of GS is a new direction for the treatment of epilepsy.

#### Purinergic Signaling

The function of astrocytes affects the occurrence of medial TLE. Dysfunction of the astrocyte signaling pathway can cause excessive excitement of the neuronal network, leading to seizures. Purinergic signal, a key signal pathway, participates in the regulation of physiological functions related to the neuron-glia signal network (169).

Adenosine triphosphate (ATP) provides energy for cell metabolism and helps to activate a variety of purinergic receptors on the cell surface to produce signal transduction. At a certain concentration, these purine receptors can affect epilepsy. Among these receptors, the metabotropic P2Y1 receptor is mainly expressed in astrocytes. A study of P2Y1 receptors showed that these receptors promote convulsions during seizures and increase the risk of spontaneous epilepsy. In addition to astrogliosis, the reactivity of microglia is an entry point for observing epilepsy, and the purine receptors of both affect the progression of epilepsy. In the hippocampus of animals, the TLR4 receptor of microglia activates the P2Y1 receptor of astrocytes, which enhances the occurrence of epileptic activity (170).

Many studies have confirmed that high concentrations of pro-inflammatory cytokines, such as tumor necrosis factor alpha (TNFα), can cause neuronal degeneration and hyperexcitability in epilepsy (171). Avignone et al. found that while the TNFα signal increased in the hippocampus after status epilepticus (SE), the P2Y1 signal was also increased (172). This indicates that the TNFα signal can lead to an increase in the purinergic signal of astrocytes, thereby inducing epilepsy. This coupling may be used as a strategy to treat epilepsy by inhibiting TNFα signaling.

## Tissue Pathology

Tissue pathology such as amygdala enlargement, tau phosphorylation, and neurocysticercosis (NCC) can be identified with the help of medical imaging facilities and histological staining.

### Amygdala Enlargement

In patients with mTLE, the most commonly identified cause of epilepsy is HS. However, the malformations of the cortex and amygdala are also observed. Many studies have found that a large proportion of mTLE patients have varying degrees of amygdala enlargement (AE). It has been speculated that AE is one of the lesions that cause mTLE, as evidenced by the diagnosis and histopathology of mTLE patients (173, 174).

Zhu et al. observed that patients with mTLE had varying degrees of AE. They collected data from three patients and found that all patients had AE of varying degrees. However, interictal epileptic discharges were observed only on the AE side (175). Pathological examination revealed abnormal development of the amygdala and temporal cortex. Six months after resection of the anterior temporal pole, amygdala, and hippocampus, none of the three patients had seizures. Researchers also found that anti-epileptic drugs have a positive effect on patients with AE. Lv et al. reported that 33 patients with AE were treated with conventional AEDs. In 67% of them, seizures did not occur and the size of the amygdala decreased (176). An increasing number of studies have confirmed that AE is one of the foci of TLE and studying the mechanism underlying it may lead to the development of TLE treatment.

### Tau Phosphorylation

Patients with TLE exhibit different degrees of cognitive decline, including the impairment of memory, language ability, executive function, attention, and reaction. Cognitive disability is greater in patients with refractory epilepsy. It is difficult to provide treatment for cognitive decline in TLE because the mechanisms underlying cognitive impairment in TLE are unknown. A link has been established between the pathology of TLE and that in Alzheimer's disease (AD): brain aging. Neurodegeneration can also contribute to cognitive dysfunction in TLE. Importantly, patients with refractory epilepsy display imaging characteristics of progressive brain aging (177), elevated amyloid-β42 burden (178), and augmented ventricular expansion (179), akin to characteristics seen in neurodegenerative cognitive disorders, such as AD.

Progressive accumulation of hyperphosphorylated tau protein in neurofibrillary tangles and amyloid-β deposition in the extracellular space form the cellular and molecular basis of cognitive decline in AD (180). These pathological changes have been observed in brain specimens from drug-resistant epilepsy cases, including patients with TLE (181) and dual-pathology epilepsies [focal cortical dysplasia (182) and brain tumor (183)]. It was found that there was a significant increase in total tau protein expression in drug-resistant epilepsy and an increase in tau phosphorylation during seizures. Neprilysin, an amyloid-β42 degrading enzyme, is significantly increased in the hippocampus (42). There is tau 5 hyperphosphorylation and overexpression at the onset of epilepsy, and overexpression of tau 5 can lead to memory decline.

Regulated by protein kinases and phosphatases, the phosphorylation of tau is a precondition for the accumulation and toxicity of tau (Figure 2D). Knocking out tau protein in a mouse epilepsy model ameliorates epileptic seizures (184). PP2A, a major tau phosphatase, can reduce the level of tau phosphorylation (185). Total PP2A level was significantly reduced in the temporal cortex of patients with drug resistant TLE (42). These studies suggest a good research direction for epilepsy caused by tau phosphorylation.

### Neurocysticercosis

Infections of the central nervous system (CNS) are common risk factors for acquired epilepsy, with viral meningitis and parasitic infections being the key causes. NCC is a central nervous system disease caused by parasites (mainly swine tapeworms) (186). In parasite-endemic countries, approximately 30% of epilepsy patients also have NCC (187). The CNS infection in NCC is caused by accidental ingestion of the improperly cooked meat containing *Taenia solium* eggs (188). This contamination caused by the deficient disposal of human feces and pigs are the intermediate hosts, while human are definitive hosts (189). When a cysticercus enters CNS, it can be detected by the immune system to induce an inflammatory response. However, in many cases, the immune response does not happen, and the host tolerates the existence of a cysticerci, leaving it undisturbed for years. Cysticerci can exist in the brain parenchyma, subarachnoid space, ventricular system, eyes, and spinal cord. Their evolution involves three stages: colloidal, granular, and calcified (190).

Patients with cysticerci in the brain parenchyma are more prone to epilepsy (191). All stages of evolution may cause seizures, but the mechanisms of epileptogenesis are different. In the colloidal stage, seizures may be due to the compression of the parenchyma and inflammation. While astrocytic gliosis surrounding the lesions is most likely the main contributor to seizures in the granular and calcified stages (192).

Hippocampal sclerosis (HS) and NCC are independent epileptic foci. Therefore, epilepsy caused by NCC and brain tissue lesions is usually called dual pathology. To confirm whether the dual pathological association between NCC and HS is accidental or causal, Mhatre et al. reviewed the cases of drug-resistant epilepsy (DRE) secondary to NCC that had been surgically removed.

After reviewing NCC cases between 2005 and 2019, researchers found that 12 patients underwent anterior temporal lobectomy and amygdalohippocampectomy (193). Neuropathological results showed that 11 of the cases were HS type 1. They also reviewed the histology of all cases and marked the tissue changes caused by cysticercosis using special stains. In the case of multiple NCC, there were traces of cysticercus in the bilateral frontal lobes, parietal lobes, and ipsilateral hippocampus. HS and NCC as independent epileptic lesions have a high frequency of dual pathology, indicating that there is a causal relationship between them. Combined resection of these two lesions is the best surgical option for the treatment of these patients.

## Conclusion

In this review, we summarized the pathological discoveries related to TLE from both animal models and human studies. We also discussed traditional pathological features, such as HS, its associated FCD, and mossy fiber sprouting. A summary of the recent pathological findings is provided, which covers gene-level, molecular level, cell-level, and tissue-level findings. We attempted to summarize many aspects of the disease. However, the review does not provide an exhaustive summary of all the findings. We hope that this review will provide an overview of the promising pathogenesis targets for TLE, which will highlight this disease to the public as well as researchers.

## Author Contributions

LL: formed the idea of the manuscript. LL and CC: revised this manuscript. JY: did the literature study. All authors wrote the manuscript.

## Conflict of Interest

The authors declare that the research was conducted in the absence of any commercial or financial relationships that could be construed as a potential conflict of interest.

## Publisher's Note

All claims expressed in this article are solely those of the authors and do not necessarily represent those of their affiliated organizations, or those of the publisher, the editors and the reviewers. Any product that may be evaluated in this article, or claim that may be made by its manufacturer, is not guaranteed or endorsed by the publisher.
